# Supplementary material for: Resolving the full spectrum of human genome variation using Linked-Reads
Source: Genome Res. 2019 Apr;29(4):635–45. doi: 10.1101/gr.234443.118 (PMC6442396; doi:10.1101/gr.234443.118)
Supplement: Supplemental Material [file supp_gr.234443.118_Supplemental_longranger-2.2.zip › longranger-2.2.tar.bz2!/lr2.2-public/lib/stan/cmdstan/stan/doc/api/html/var__stack_8hpp.html]

Stan: src/stan/agrad/rev/var\_stack.hpp File Reference


|  |  |
| --- | --- |
|  | Stan  2.5.0  probability, sampling & optimization |


- Main Page
- Related Pages
- Namespaces
- Classes
- Files

- File List
- File Members

All Classes Namespaces Files Functions Variables Typedefs Enumerations Enumerator Friends Macros Pages

- src
- stan
- agrad
- rev

Classes |
Namespaces |
Functions

var\_stack.hpp File Reference

`#include <stdexcept>`  
`#include <vector>`  
`#include <stan/memory/stack_alloc.hpp>`

Go to the source code of this file.

|  |  |
| --- | --- |
| Classes | |
| class | stan::agrad::chainable\_alloc |
|  | A chainable\_alloc is an object which is constructed and destructed normally but the memory lifespan is managed along with the arena allocator for the gradient calculation. More... |
|  | |

|  |  |
| --- | --- |
| Namespaces | |
|  | stan |
|  | Probability, optimization and sampling library. |
|  | |
|  | stan::agrad |
|  | Function gradients via reverse-mode automatic differentiation. |
|  | |

|  |  |
| --- | --- |
| Functions | |
| static bool | stan::agrad::empty\_nested () |
|  | Return true if there is no nested autodiff being executed. More... |
|  | |
| static void | stan::agrad::recover\_memory () |
|  | Recover memory used for all variables for reuse. More... |
|  | |
| static void | stan::agrad::recover\_memory\_nested () |
|  | Recover only the memory used for the top nested call. More... |
|  | |
| static void | stan::agrad::start\_nested () |
|  | Record the current position so that `recover_memory_nested()` can find it. More... |
|  | |
| static size\_t | stan::agrad::nested\_size () |
|  | |


---

[ Stan Home Page ]

*© 2011–2014,
Stan Development Team.*
